# CD4 T Cell-Derived IFN-γ Plays a Minimal Role in Control of Pulmonary *Mycobacterium tuberculosis* Infection and Must Be Actively Repressed by PD-1 to Prevent Lethal Disease

**DOI:** 10.1371/journal.ppat.1005667

**Published:** 2016-05-31

**Authors:** Shunsuke Sakai, Keith D. Kauffman, Michelle A. Sallin, Arlene H. Sharpe, Howard A. Young, Vitaly V. Ganusov, Daniel L. Barber

**Affiliations:** 1 T lymphocyte Biology Unit, Laboratory of Parasitic Diseases, National Institute of Allergy and Infectious Diseases, National Institutes of Health, Bethesda, Maryland, United States of America; 2 Department of Microbiology and Immunobiology, and Evergrande Center for Immunological Diseases, Harvard Medical School and Brigham and Women’s Hospital, Boston, Massachusetts, United States of America; 3 Cancer and Inflammation Program, National Cancer Institute, Frederick, Maryland, United States of America; 4 Department of Microbiology, University of Tennessee, Knoxville, Tennessee, United States of America; Harvard School of Public Health, UNITED STATES

## Abstract

IFN-γ–producing CD4 T cells are required for protection against *Mycobacterium tuberculosis* (Mtb) infection, but the extent to which IFN-γ contributes to overall CD4 T cell-mediated protection remains unclear. Furthermore, it is not known if increasing IFN-γ production by CD4 T cells is desirable in Mtb infection. Here we show that IFN-γ accounts for only ~30% of CD4 T cell-dependent cumulative bacterial control in the lungs over the first six weeks of infection, but >80% of control in the spleen. Moreover, increasing the IFN-γ–producing capacity of CD4 T cells by ~2 fold exacerbates lung infection and leads to the early death of the host, despite enhancing control in the spleen. In addition, we show that the inhibitory receptor PD-1 facilitates host resistance to Mtb by preventing the detrimental over-production of IFN-γ by CD4 T cells. Specifically, PD-1 suppressed the parenchymal accumulation of and pathogenic IFN-γ production by the CXCR3^+^KLRG1^-^CX3CR1^-^ subset of lung-homing CD4 T cells that otherwise mediates control of Mtb infection. Therefore, the primary role for T cell-derived IFN-γ in Mtb infection is at extra-pulmonary sites, and the host-protective subset of CD4 T cells requires negative regulation of IFN-γ production by PD-1 to prevent lethal immune-mediated pathology.

## Author Summary

The development of novel tuberculosis vaccines has been hindered by the poor understanding of the mechanisms of host-protection. It has been long-held that IFN-γ is the principle effector of CD4 T cell-mediated resistance to Mtb infection, but Mtb-specific CD4 T cells produce low amounts of IFN-γ in vivo, leading to the possibility that increasing IFN-γ production by Th1 cells might enhance control of Mtb infection. However, the precise contribution of IFN-γ to CD4 T cell-dependent protection and the outcome of increasing IFN-γ production by CD4 T cells have not been evaluated. Here we show that IFN-γ accounts for only ~30% of the cumulative CD4 T cell-mediated reduction in lung bacterial loads over the first 1.5 months of infection. Moreover, we find that increasing the per capita production of IFN-γ by CD4 T cells leads to the early death of the host. Lastly, we show that suppression of CD4 T cell-derived IFN-γ by the inhibitory receptor PD-1 is essential to prevent lethal disease. Therefore, poor control Mtb infection does not result from defective production of IFN-γ, and strategies to selectively boost it are unwarranted. Furthermore, identifying the primary mechanisms of CD4 T cell-dependent control of Mtb infection should be a priority.

## Introduction

*Mycobacterium tuberculosis* (Mtb) infection is a leading cause of global morbidity and mortality. In 2014 there were 9.6 million new cases of tuberculosis (TB) and 1.5 million deaths resulting from Mtb infection [1]. The only available vaccine against Mtb infection, Bacillus Calmette-Guérin (BCG), is an attenuated strain of *M*. *bovis* that was developed almost a century ago. BCG immunization does prevent severe forms of childhood TB but at best poorly protects against adult disease [2] It is widely accepted that effective vaccination approaches for TB would have an enormous impact on global health; however, efforts in TB vaccine development have been hindered by the lack of mechanistic insight into the cellular and molecular basis of both protective immunity and immunopathology during TB.

CD4 T cells are essential for host resistance to Mtb infection [3] and the protection afforded by various vaccination approaches in experimental animal models is mediated mainly by CD4 T cells [4–8]. Although other cell types may make contributions to vaccine-elicited protection against Mtb infection, it seems likely that a successful vaccination strategy will require the induction of MHC class II-restricted CD4 T cell responses of the sufficient quantity, location, breadth of specificity, and polarized effector capacity. IFN-γ is a key CD4 T cell-derived cytokine and essential for resistance to mycobacterial infections. Mice deficient in IL-12, T-bet, or IFN-γ itself are extremely susceptible to Mtb infection [9]. Humans with inborn errors in the IFN-γ axis are highly susceptible to normally avirulent non-tuberculous mycobacterial (NTM) infections. Deficiencies in IL-12p40, IL-12RI or II, IFN-γR or STAT-1 [10, 11] all result in severe NTM infections early in life. Adults who develop anti-IFN-γ neutralizing autoantibodies are also very prone to mycobacterial infections later in life [12]. Due to the severity of infection in its absence, IFN-γ is often considered the primary mechanism by which the host controls Mtb infection. Although several immune cell types can produce IFN-γ, it has been shown that IFN-γ must be produced by CD4 T cells for the host to survive Mtb infection [13]. However, IFN-γ responses do not correlate with better outcome of Mtb infection [14–16], and a recent vaccine efficacy trial based on a viral vector containing an Mtb antigen was found to generate bacilli-specific CD4 T cells capable of producing high levels of IFN-γ but afforded no protection against the development of TB [17]. It remains unclear why IFN-γ responses are not observed to correlate with resistance to Mtb infection. This may suggest that very small amounts of IFN-γ are needed for optimal protection, or that IFN-γ may even be associated with disease rather than protection during active TB [18]. Also, these findings could indicate that other T cell effector molecules are the primary mediators of bacterial control.

Several studies have found that the amount of IFN-γ produced by Mtb-specific [19, 20] or BCG-specific [21] CD4 T cells in vivo is much lower than expected based on their ability to produce IFN-γ after stimulation with high doses of peptide, with usually <10% of the cells producing IFN-γ in the infected tissue. This has led to the hypotheses that defective production of IFN-γ by Mtb-specific CD4 T cells contributes to the host’s inability to control Mtb. This poor IFN-γ production by Mtb-specific CD4 T cells is at least in part due to poor antigen (Ag) presentation, as these studies have found that increasing Ag concentrations in the tissue enhanced IFN-γ production by CD4 T cells in vivo, and Mtb has evolved mechanisms that interfere with Ag-presentation [22]. The low IFN-γ production may also reflect the degree of effector polarization of the CD4 T cells. Based on several studies, it is now clear that Mtb-specific CD4 T cells that have migrated into the lung tissue parenchyma of Mtb-infected mice (marked by CXCR3 expression) express lower amounts of the key transcription factor T-bet and produce less IFN-γ compared to the KLRG1^+^CX3CR1^+^ CD4 T cells that localize to the lung-associated blood vasculature [20] [23] [24] [25] [26]. Despite their reduced IFN-γ producing capacity, this population of CXCR3^+^KLRG1^-^CX3CR1^-^ CD4 T cells displayed the best protective capacity against Mtb infection, likely due to their ability to migrate into the lung tissue and interact with infected myeloid cells within the granulomas. Indeed, direct interactions between CD4 T cells and MHCII molecules on infected macrophages is required for the optimal suppression of intracellular growth of Mtb [27]. T cell dysfunction in the form of exhaustion or adaptive tolerance could also play a role in the reduced IFN-γ production by Mtb-specific CD4 T cells. The lung-homing, host-protective population of CD4 T cells expresses high levels of multiple co-inhibitory receptors including programmed cell death-1 (PD-1) [23, 24], which is well known to suppress IFN-γ production [28] [29]. The lungs of Mtb-infected PD-1 KO mice contain greatly increased concentrations of IFN-γ compared to WT mice [30, 31]. However, rather than displaying enhanced control of Mtb, these animals succumb due to CD4 T cell-mediated immunopathology, although precise mechanisms leading to the death of PD-1 KO mice have not been identified. Therefore, there is reason to suggest that defective IFN-γ production by CD4 T cells could account for poor control of Mtb, but also data to indicate that increased IFN-γ production by CD4 T cells might be detrimental under some circumstances.

These observations prompted us to ask several questions regarding the role of CD4 T cell-derived IFN-γ in Mtb infection. First, what is the relative contribution of IFN-γ production to total CD4 T cell-mediated control of Mtb infection? Second, is the inability of the host to better control Mtb infection due to the inadequate production of IFN-γ by the lung parenchymal CD4 T cells? Third, does selectively enhancing the production of IFN-γ by CD4 T cells result in better control of Mtb infection? Lastly, what is the role of PD-1-mediated inhibition of IFN-γ production by Mtb-specific CD4 T cells in protection and pathology? We found that the relative contribution of CD4 T cell-derived IFN-γ to the control of Mtb infection varies dramatically by tissue, with surprisingly small contribution in the lungs compared to the spleen. The amount of IFN-γ production by pulmonary CD4 T cells is more than sufficient for optimal bacterial control in the lung, and increasing the amount of IFN-γ that individual CD4 T cells produce leads to lethal disease. Moreover, we found that host survival of Mtb infection requires PD-1 mediated inhibition of IFN-γ production by parenchymal CXCR3^+^CX3CR1^-^KLRG1^-^ effector CD4 T cells, which mediate the best protection against Mtb infection. Therefore, the inability of the host to control Mtb infection does not result from poor IFN-γ production, and selectively increasing IFN-γ production on a per cell basis is detrimental to the outcome of pulmonary infection. These data refine our view of the role of IFN-γ, which is classically considered the primary determinant of anti-mycobacterial immunity, and provide insight into the role of T cell co-inhibition in Mtb infection. Moreover, they show that the same CD4 T cells that mediate best host resistance to Mtb infection are also the most capable of driving lethal disease if not properly regulated.

## Results

### CD4 T cell-derived IFN-γ contributes relatively little to control of Mtb infection in the lung but is essential in the spleen

**Fig 1:**
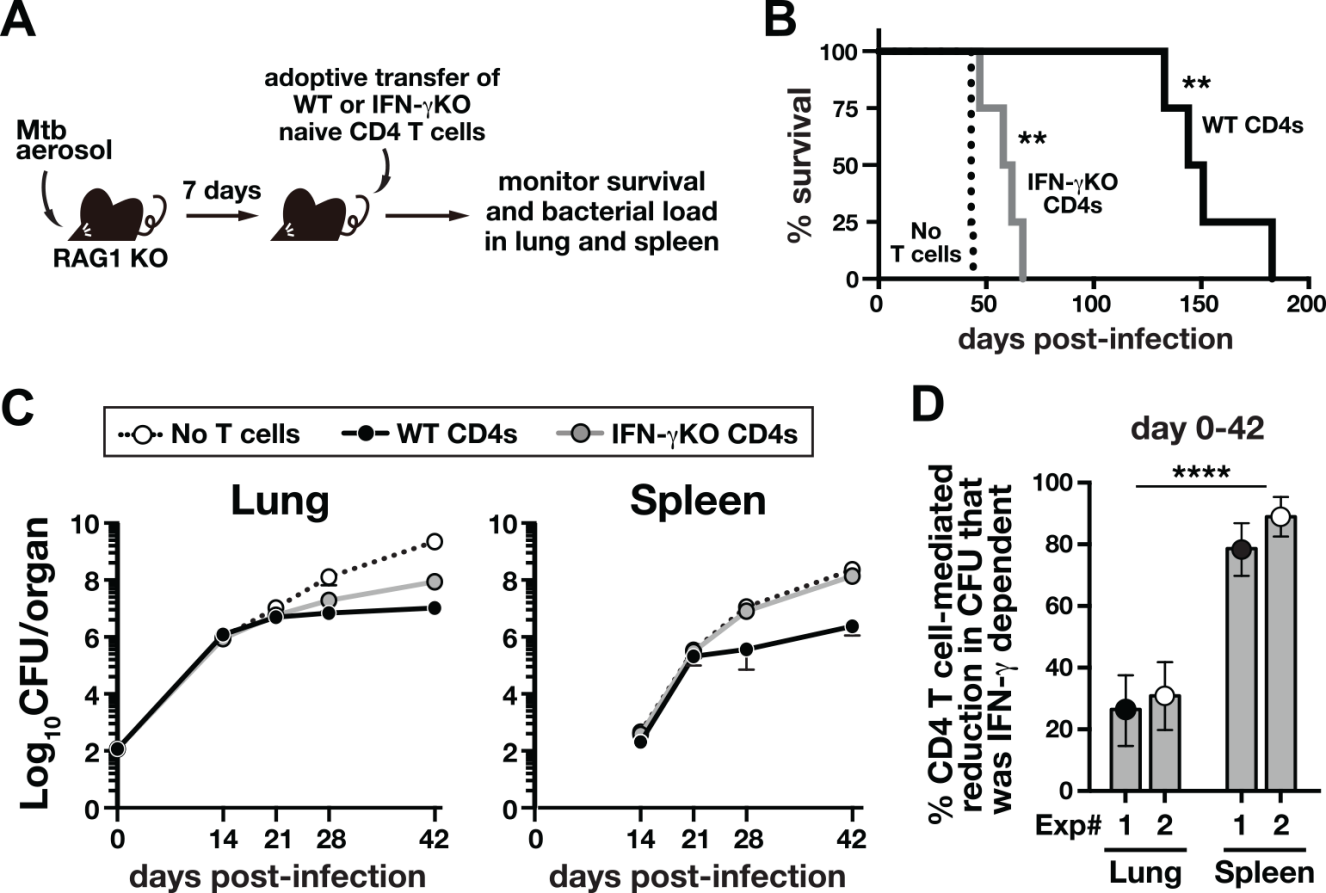
CD4 T cell-derived IFN-γ contributes little to control of Mtb infection in the lung but is essential in the spleen. (**A-C**) Naïve CD4 T cells isolated from either WT or IFN-γ KO mice were adoptively transferred into day-7 infected RAG1 KO recipients (**A**), and survival of mice (**B**) and bacterial load in the tissues (**C**) were monitored. Data are representative of two independent experiments. (n = 4-5/group/experiment). **, *P*<0.005. (**D**) The areas between the log transformed bacterial growth curves in untreated control mice and WT CD4 T cell recipients or control mice and IFN-γ KO CD4 T cell recipients shown in (**C**) were calculated for the lungs and spleens. The ratio of IFN-γ KO:WT CD4 T cell areas was then calculated to estimate the proportion of cumulative bacterial reductions that were due to CD4 T cell-derived IFN-γ. Each dot represents the result obtained from two independent experiments. Data are mean ± 95% confidence interval. ****, *P*<0.0001 (bootstrap test).

To examine the role of CD4 T cell-derived IFN-γ in host survival and bacterial control in different tissues, we performed a series of adoptive transfer experiments using RAG1 KO recipients (Fig 1A). Transfer of WT CD4 T cells protected Mtb infected RAG1 KO hosts for >130 days (Fig 1B). In contrast, reconstitution with IFN-γ KO CD4 T cells only slightly extended survival compared to recipients that did not receive T cells (mean survival time, 60 versus 43 days, respectively; Fig 1A and 1B). Thus, production of IFN-γ by CD4 T cell is required for host survival of Mtb infection, consistent with several previous reports [13, 32].

The relative survival of mice containing WT or IFN-γ KO CD4 T cells, however, does not provide quantitative information on the importance of CD4 T cell-derived IFN-γ to bacterial control. We next sought to estimate the relative contribution of IFN-γ to the overall CD4 T cell-mediated suppression of Mtb growth in vivo. To do so, we utilized a similar adoptive transfer model and monitored bacterial loads in the lungs and spleens until 42 days post-infection (p.i.), after which unreconstituted RAG1 KO mice succumb and further comparisons with T cell reconstituted mice cannot be made. Compared to WT CD4 T cells, IFN-γ KO CD4 T cells were able to suppress Mtb replication far better in the lungs compared to the spleen (Fig 1C). Using a simple mathematical model we showed that by comparing the area under the log transformed bacterial growth curves in control RAG1 KO mice vs. recipients of IFN-γ KO or WT CD4 T cells, we could estimate the relative contribution of CD4 T cell-derived IFN-γ to the overall CD4 T cell-mediated control of Mtb growth in the lungs and spleens (see S1 Method for description of the mathematical model). By performing these calculations for two independent experiments, we found that CD4 T cell-derived IFN-γ accounted for ~30% of CD4 T cell-dependent bacterial control in the lungs between day 0 to 42 p.i., but ~80% in the spleens (Fig 1D). Based on our calculation, CD4 T cell-derived IFN-γ contributes surprisingly little to control of pulmonary Mtb infection, but is the principal protective effector molecule in the spleen, at least during the first 6 weeks post-exposure.

### IFN-γ producing CD4 T cells are not limiting, and increasing the production of IFN-γ by CD4 T cells leads to the early mortality of host

**Fig 2:**
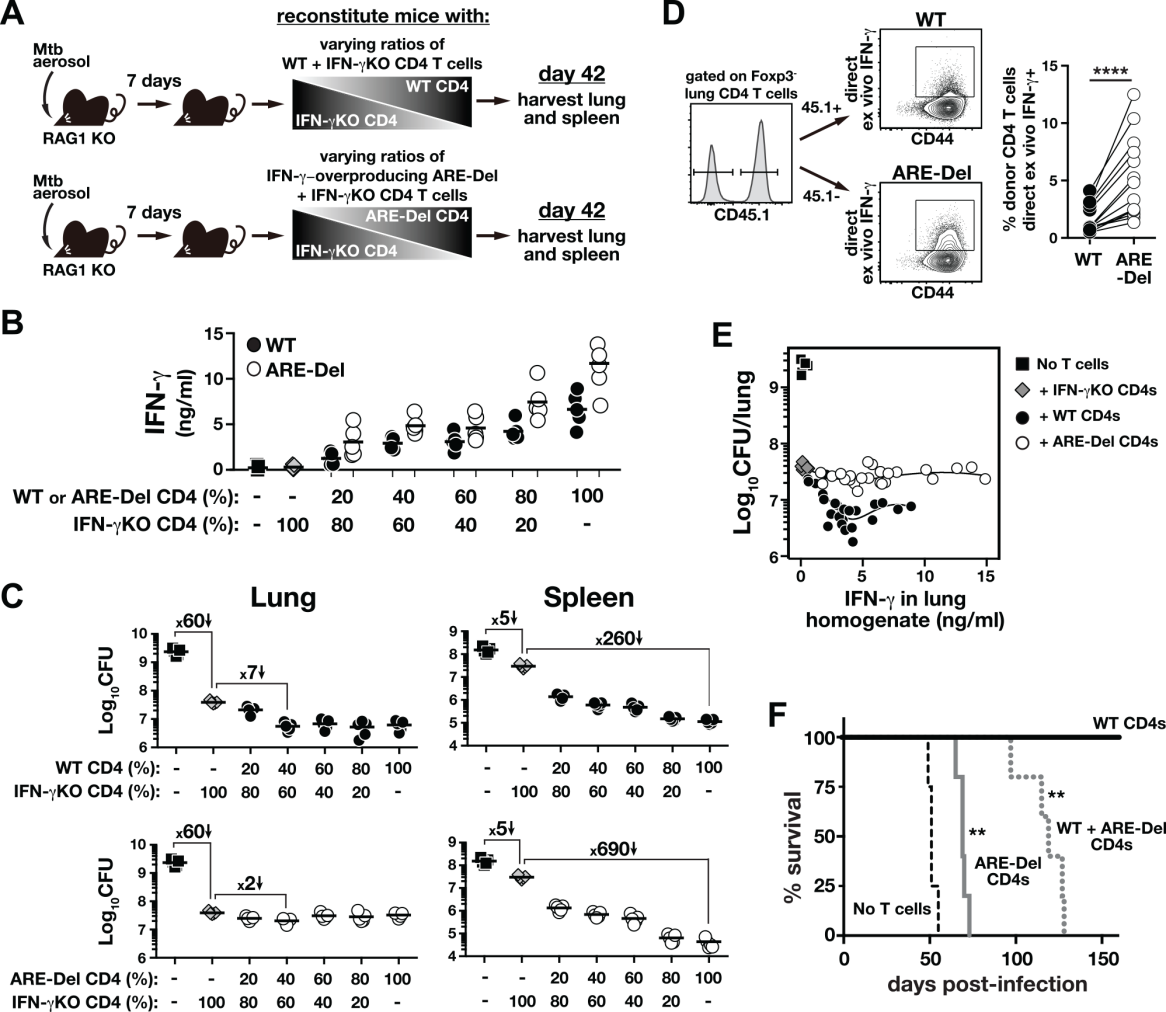
Increased IFN-γ–production by CD4 T cells exacerbates pulmonary Mtb infection and leads to the early host mortality, despite enhancing bacterial control in the spleen. RAG1 KO mice were infected with Mtb 7 days earlier and reconstituted with CD4 T cells from uninfected donors at increasing ratios of either WT or ARE-Del CD4 T cells mixed with IFN-γ KO CD4 T cells (**A**). All mice received the same total number of donor CD4 T cells, as only the fractions of IFN-γ–producing CD4 T cells varied (either WT or IFN-γ–overproducing). On day 42 IFN-γ concentrations in the lung homogenates (**B**) and bacterial load in the tissues were measured (**C**). Data are representative of two independent experiments (n = 5/group/experiment). (**D**) A mixture of WT and ARE-Del CD4 T cells (at 1:1 ratio) were co-transferred into day-7 infected TCRα KO mice, and on day 60 IFN-γ production by donor CD4 T cells was measured by DrxICS. Data are pooled from two independent experiments (n = 6/experiment) and each connecting line represents an individual animal. ****, *P*<0.0001 (**E**) Correlation between IFN-γ levels and bacterial numbers in the lungs of RAG1 KO mice. Data shown are replotted from the values shown in (**B and C**) to illustrate the correlation. (**F**) TCRα KO mice were infected with Mtb 7 days before and received with WT, ARE-Del or a 1:1 mixture of WT and ARE-Del naïve CD4 T cells and mouse survival was monitored. Data are representative of three independent experiments. (n = 4-5/group/experiment). **, *P*<0.002; compared to control group received WT CD4 T cells alone.

The relatively minor contribution of CD4 T cell-derived IFN-γ to control of Mtb in the lungs might be explained by insufficient production of this cytokine by pulmonary T cells [24]. To test this hypothesis, we asked if the amount of IFN-γ produced by CD4 T cells is below what is needed to achieve maximum levels of CD4 T cell-mediated bacterial control in the tissues. To this end, RAG1 KO mice infected with Mtb 7 days prior were reconstituted with naïve CD4 T cells (3 × 10^6^ cells/recipient) at varying ratios of WT to IFN-γ KO CD4 T cells, and on day 42 p.i. lungs and spleens were harvested (Fig 2A). We found that IFN-γ concentrations in the lung homogenates rose steadily as the proportion of WT CD4 T cells in the mixed inoculum was increased (ranging from ~100 pg/ml to ~8 ng/ml; Fig 2B). Mice reconstituted with IFN-γ KO CD4 T cells alone showed a ~60-fold decrease in CFU in the lungs compared to the group not receiving cells (Fig 2C, *upper left*). As the proportion of WT T cells to IFN-γ KO cells was increased, the bacterial loads in the lungs further decreased ~7-fold. Importantly, the CFU reached a nadir at 40% WT cells, and although the concentrations of IFN-γ continued to rise as more IFN-γ–producing T cells were introduced, the bacterial loads did not further decrease. In fact, mice that received 100% WT CD4 T cells had the same CFU counts in their lungs as mice that received 40% WT + 60% KO T cells. In the spleen, adoptive transfer of IFN-γ KO CD4 T cells alone into RAG1 KO mice resulted in only ~5-fold reduction in CFU compared to the control mice that did not receive cells (Fig 2C, *upper right*). In contrast to the lungs, the bacterial loads in the spleens continued to decrease as the proportion of WT CD4 T cells increased, reaching a nadir of ~260-fold reduction at 100% WT compared to 100% IFN-γ KO CD4 T cells. Therefore, these data support our previous results showing a tissue-specific role for IFN-γ (Fig 1). Moreover, these data suggest that the limited contribution of CD4 T cell-derived IFN-γ in the lungs cannot be explained by its insufficient production.

To further test the hypothesis that defective IFN-γ production by CD4 T cells does not limit the effectiveness of Mtb-specific CD4 T cells, we performed the in vivo titration experiment with mixtures of IFN-γ–overproducing CD4 T cells and IFN-γ KO CD4 T cells. To do so, we employed CD4 T cells in which the AU-rich element (ARE) in the 3’ untranslated region of the IFN-γ gene is deleted (ARE-Del), resulting in stabilization of the IFN-γ mRNA and increased IFN-γ translation [33]. The ARE-Del CD4 T cells produced ~2-fold higher amounts of IFN-γ in the lung compared to WT CD4 T cells at each incremental ratio (Fig 2B). We confirmed the increased expression of IFN-γ in ARE-Del CD4 T cells by direct ex vivo intracellular staining (DrxICS) in the mice that were received a 1:1 mixture of WT and ARE-Del CD4 T cells (Fig 2D). By this flow cytometry-based assay, there was again ~2 fold increase in IFN-γ expressing CD4 T cells in the ARE-Del compared to WT CD4 T cells. We found an approximately 1.1 fold increase in the geometricMFI of IFN-γ staining in the ARE-Del CD4 T cells compared to WT counterparts in the same lung (1.06 ± 0.01, *P* = 0.02), but due to the very low staining and lack of separation from the negative cells, inherent in the approach, we cannot be certain of the precise increase in the amount of cytokine produced per cell in the ARE-Del CD4 T cells. Strikingly, IFN-γ–over-producing ARE-Del CD4 T cells were less effective at controlling Mtb growth in the lungs compared to WT CD4 T cells. As IFN-γ–over-expressing CD4 T cells were titrated into the system, CFU counts reached a nadir of ~2 fold reduction (below the CFU amounts found at 100% IFN-γ KO CD4 T cells) at a ratio of 40% ARE-Del to 60% KO CD4 T cells (Fig 2C). As the fraction of ARE-Del CD4 T cells was increased further, the CFU began to rise slightly, so that the levels of bacteria in the lungs of mice that received 100% IFN-γ KO CD4 T cells and 100% ARE-Del CD4 T cells were identical, despite the enormous difference in IFN-γ concentrations in the lung. In the spleens however, the IFN-γ–overproducing CD4 T cells were more effective than WT CD4 T cells at controlling Mtb growth (Fig 2C). Mice receiving 100% ARE-Del CD4 T cells displayed a ~690-fold reduction in bacterial loads in their spleens compared to recipients of 100% IFN-γ KO CD4 T cells versus the 260 fold reduction seen in WT CD4 T cell recipients, indicating that impressive levels of bacterial control can be achieved in the spleen when IFN-γ production by CD4 T cells is amplified beyond normal levels. This experiment allowed us to examine the relationship between IFN-γ in the lung tissues and lung bacterial loads in the setting of WT and elevated IFN-γ production by CD4 T cells (Fig 2E). With WT CD4 T cells, there was a negative relationship between IFN-γ concentrations and CFU counts in the lung that levels off at ~4 ng/ml IFN-γ in the lungs in this particular experimental setting. In contrast, with IFN-γ–overproducing CD4 T cells this negative correlation was lost. These data highlight how the impact of IFN-γ concentrations on bacterial loads depends more on the amount of the cytokine produced per T cell rather than the total amount found in the tissue. Moreover, increasing IFN-γ production by CD4 T cells not only failed to enhance suppression of Mtb growth in the lungs, but actually impaired control.

To examine the outcome of increased IFN-γ production by CD4 T cells on host survival of Mtb infection, we reconstituted Mtb-infected TCRα KO mice with WT, ARE-Del or a 1:1 mixture of WT and ARE-Del CD4 T cells and monitored mouse survival. Mice that did not receive cells all succumbed by day 60 p.i., whereas mice reconstituted with WT CD4 T cells all survived for >150 days (Fig 2F). Notably, mice reconstituted with ARE-Del CD4 T cells all succumbed by day 80 p.i. This increased susceptibility was observed even in the presence of WT CD4 T cells, indicating that ARE-Del CD4 T cells actively promote early mortality after Mtb infection. Collectively, these data show that the inability of the host to control Mtb infection in the lungs does not result from inadequate production of IFN-γ, and increased IFN-γ production by CD4 T cells is, in fact, detrimental to control pulmonary infection and host survival of Mtb infection. In marked contrast, control of Mtb infection in the spleen is highly dependent on CD4 T cell-derived IFN-γ and growth of Mtb is tightly linked to the levels of IFN-γ produced by splenic T cells.

### CD4 T cell-derived TNF has similarly minor contributions to control of Mtb infection in the lung and spleen

**Fig 3:**
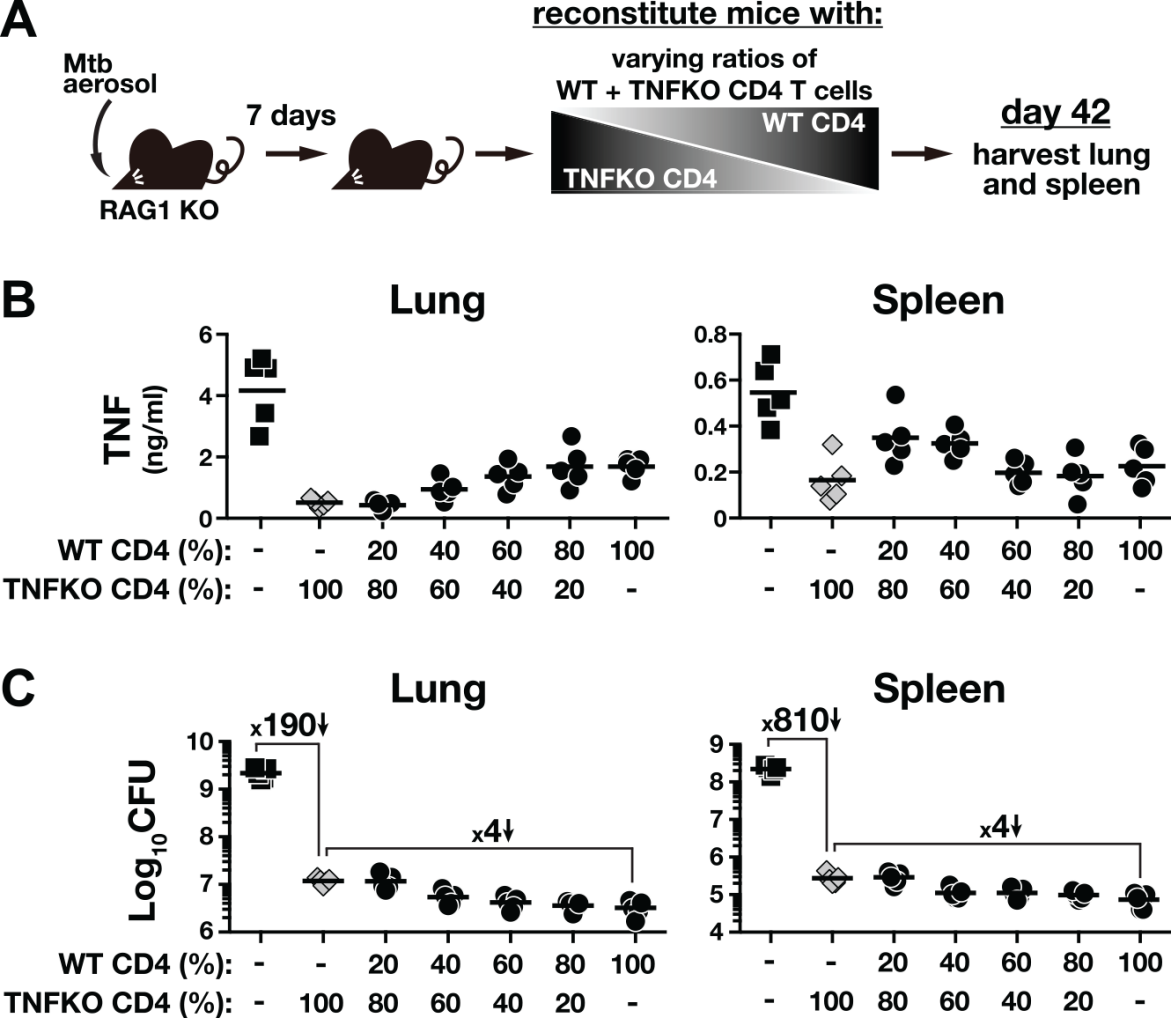
CD4 T cell-derived TNF has a minor contribution to inhibition of Mtb growth in both lung and spleen. CD4 T cells isolated from naïve WT or TNF KO mice were adoptively transferred into day-7 infected RAG1 KO recipients at increasing ratios of WT cells to TNF KO cells. All mice received the same total number of donor CD4 T cells, as only the fractions of TNF–producing CD4 T cells varied (**A**). TNF levels in the tissue homogenates (**B**) and bacterial load in the tissues (**C**) were measured on day 42 p.i. Data are representative of two independent experiments (n = 5/group/experiment).

TNF is critical for host resistance to TB [34], and TNF production by CD4 T cells is required for the long-term control of Mtb infection [35] [36] [37]. Given the limited role for CD4 T cell-derived IFN-γ in control Mtb infection in the lung, we next asked if TNF could account for the majority of CD4 T cell-dependent pulmonary bacterial control. To do so, we performed similar in vivo titration experiments with WT and TNF KO CD4 T cells (Fig 3A). We observed that TNF concentrations were the highest in the both lungs and spleens of RAG1 KO mice that did not receive cells and that reconstitution of RAG1 KO mice with 100% TNF KO CD4 T cells leads to a decrease in TNF levels in the lung and spleen homogenates (Fig 3B). Mice that received 100% TNF KO CD4 T cells also displayed a ~190 fold reduction in bacterial loads in the lungs and ~810 fold reduction in the spleens (Fig 3C). Therefore, TNF KO CD4 T cells were able to dramatically restrict bacterial replication in both tissues, likely explaining the decreased amounts of non-T cell derived-TNF in the tissue homogenates. As the proportion of WT cells in the transferred cell population was increased, the levels of TNF in the lung and spleen homogenates gradually rose (Fig 3B). In the spleens, however, increasing the percentage of the T cell inoculum above 40% WT resulted in a decline in total TNF concentrations. Importantly, as the proportion of WT CD4 T cells in the inoculum was increased, the bacterial loads decreased an additional ~ 4 fold below what was observed with 100% TNF KO CD4 T cells in both the lungs and spleens (Fig 3C, *left*). Therefore, we did not observe a tissue-specific role for CD4 T cell-derived TNF in control of Mtb infection as we did for IFN-γ. These data also argue that TNF is not the major contributor to CD4 T cell-dependent control of Mtb infection in the lung.

### PD-1 suppresses the accumulation of parenchymal CD4 T cells and their IFN-γ production in Mtb infection

**Fig 4:**
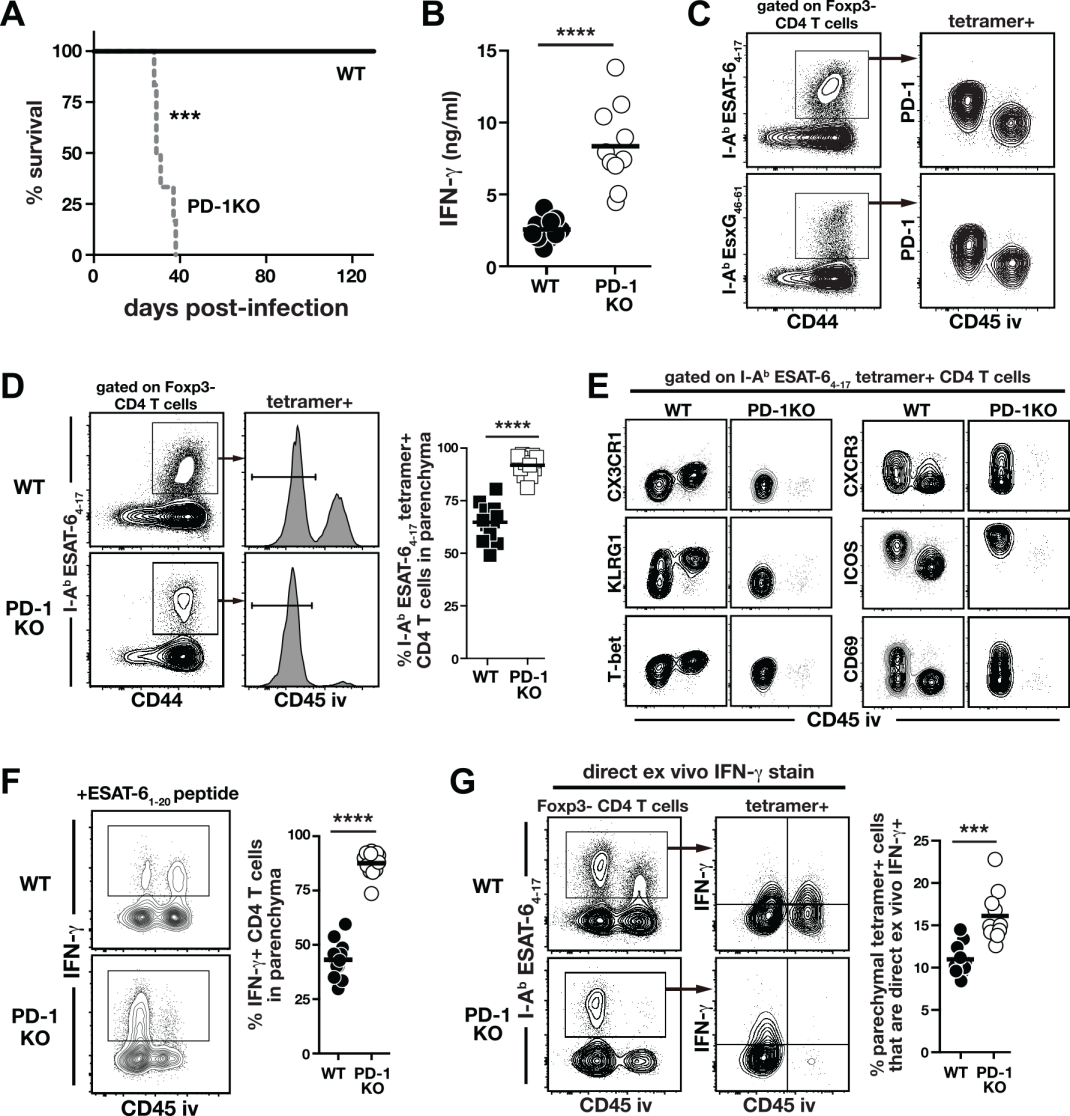
Mtb-specific CD4 T cells in the lungs of PD-1 KO mice are less differentiated, more parenchymally localized and produce increased amounts of IFN-γ. (**A**) Survival of WT and PD-1 KO mice after Mtb infection. Data are representative of at least three independent experiments. (n = 5-6/experiment). (**B**) IFN-γ levels in the lung homogenates of WT and PD-1 KO mice on day 30 p.i. Data are pooled from three independent experiments (n = 3-4/experiment). (**C**) PD-1 expression on the parenchymal and intravascular I-A^b^ESAT-6_4–17_ or I-A^b^EsxG_46–61_–specific CD4 T cells in WT lung on day 30 p.i. (**D**) Iv-staining of I-A^b^ESAT-6_4–17_–specific CD4 T cells in the lung of WT and PD-1 KO mice on day 30 p.i. Data are pooled from three independent experiments (n = 3-4/experiment). (**E**) Phenotypic analysis of I-A^b^ESAT-6_4–17_–specific CD4 T cells in WT and PD-1 KO lungs on day 30 p.i. (**F**) Intracellular IFN-γ staining of lung CD4 T cells in WT and PD-1 KO mice after in vitro stimulation with ESAT-6_1–20_ peptide on day 30 p.i. Data are pooled from three independent experiments (n = 3-4/experiment). (**G**) Direct ex vivo IFN-γ staining for I-A^b^ESAT-6_4–17_–specific CD4 T cells in the lungs of WT and PD-1 KO mice on day 30 p.i. Data are pooled from three independent experiments (n = 3-4/experiment). Cells in (**A**) and (**E**) were pooled from n = 3/experiment for FACS analysis. ***, *P*<0.0005; ****, *P*<0.0001.

Given that IFN-γ makes relatively little contribution to control of pulmonary Mtb infection and that artificial over-expression of IFN-γ promotes disease, we next considered the possibility that IFN-γ production by CD4 T cells must be appropriately suppressed, not enhanced, for host-resistance to Mtb infection. The co-inhibitory receptor PD-1 is well understood to suppress IFN-γ production by T cells [28, 29], and PD-1 KO mice are extremely susceptible to Mtb infection [30, 31, 38] due to CD4 T cell-driven disease [31]. Here we confirmed that PD-1 KO mice succumb rapidly to infection with high levels of IFN-γ in their lungs (Fig 4A and 4B). Moreover, using an intravascular stain (iv-stain) technique that allows discrimination between intravascular and parenchymal T cells [39], we confirmed that I-A^b^ESAT-6_4−17_ and I-A^b^EsxG_46-61_-specific CD4 T cells in the lung tissue parenchyma express higher levels of PD-1 than their iv counterparts ([24]; Fig 4C). We next used the iv-stain approach to determine the impact of PD-1 deficiency on the distribution of Mtb-specific CD4 T cells between the lung parenchyma and lung-associated blood vasculature. PD-1 KO mice showed a remarkable increase in accumulation of I-A^b^ESAT-6_4–17_–specific CD4 T cells in the lung parenchyma compared to WT mice (~95% versus ~60%, respectively; Fig 4D). The increased accumulation of lung parenchymal CD4 T cells in the PD-1 KO mice was associated with loss of markers we have previously shown to identify intravascular CD4 T cells (i.e., CX3CR1^+^, KLRG1^+^ and T-bet^high^) and increased expression of makers that are associated with the protective parenchymal cells including CXCR3 and ICOS but decreased level of CD69 ([20, 24]; Fig 4E). We next compared the ability of lung Mtb-specific CD4 T cells to produce IFN-γ upon in vitro stimulation with ESAT-6_1–20_ peptide, and observed a marked increase in lung parenchymal localization of IFN-γ–producing CD4 T cells in PD-1 KO mice compared to WT mice (~90% versus ~40%, respectively; Fig 4F). To evaluate the amount of IFN-γ production by WT and KO Mtb-specific CD4 T cells in vivo, IFN-γ expression by I-A^b^ESAT-6_4–17_–specific CD4 T cells was measured by DrxICS. We found that ~2-fold more parenchymal tetramer^+^ PD-1 KO CD4 T cells actively expressed IFN-γ in vivo compared to WT counterparts (Fig 4G). These data show that during Mtb infection PD-1 suppresses the generation of CD4 T cells with the lung-homing phenotype as well as the production of IFN-γ by the CD4 T cells that have migrated into the lung parenchyma.

**Fig 5:**
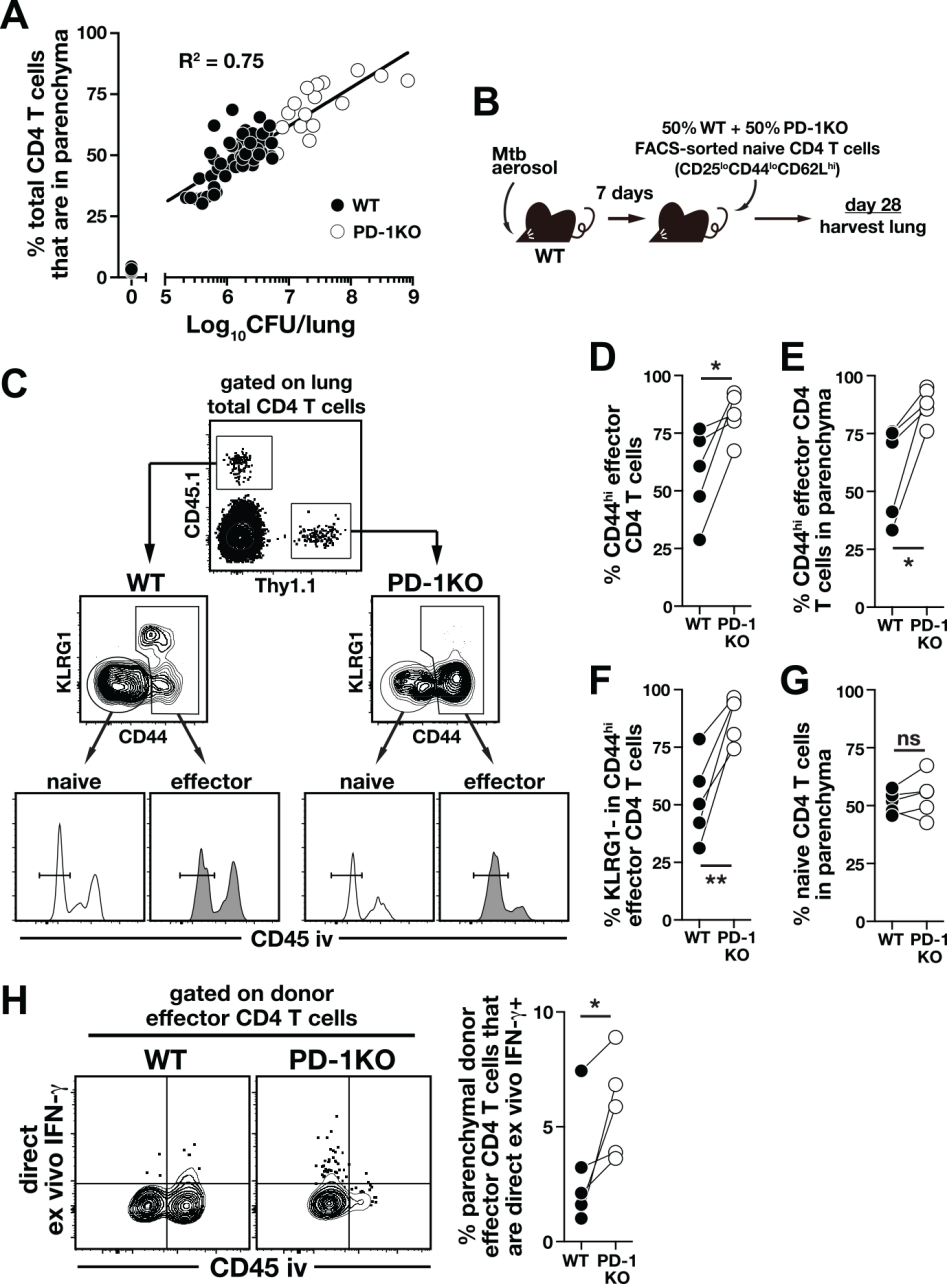
PD-1 expression on CD4 T cells inhibits accumulation of and IFN-γ production by lung parenchymal CD4 T cells during Mtb infection. (**A**) Correlation between bacterial load and frequency of parenchymal CD4 T cells in the lungs of Mtb infected mice. Data are pooled from experiments performed at different times p.i. ranging from day 0 to day 180. (**B-F**) FACS purified naïve CD4 T cells from WT (CD45.1) and PD-1 KO (Thy1.1) mice were mixed at a 1:1 ratio and co-transferred into day-7 infected WT mice **(B)**. On day 28 an iv-stain was performed in the recipient mice and donor CD4 T cells were identified by their congenic markers (**C**). The frequency of donor total CD44^hi^ effector CD4 T cells in the recipient lungs (**D**) and in the lung parenchyma (**E**). (**F**) The frequency of KLRG1^-^ cells in the donor CD44^hi^ effector CD4 T cells. (**G**) The frequency of donor naïve CD4 T cells accumulating in the lung parenchyma. (**H**) IFN-γ production of donor effector CD4 T cells was determined by DrxICS. Data are representative of two independent experiments (n = 5/experiment) and each connecting line represents an individual mouse (n = 5/experiment). *, *P*<0.02; **, *P*<0.006.

Although our data suggest that PD-1 has a role in regulating Mtb-specific CD4 T cell responses in the lung parenchyma, we also found a positive correlation between the bacterial load in the lungs and the frequency of lung parenchymal CD4 T cells (Fig 5A). Thus, the increase in CD4 T cell accumulation and IFN-γ production in the lung parenchyma might be secondary to the higher bacterial burden in the lungs of PD-1 mice (Fig 4D and 4F) rather than to a direct effect of the loss of PD-1. To examine the cell-intrinsic role of PD-1 in CD4 T cells, pure naïve (CD25^lo^CD44^lo^CD62L^hi^) CD4 T cells were sorted (to >97% purity) from WT or PD-1 KO mice, mixed at 1:1 ratio, and transferred into Mtb-infected congenically disparate WT recipient mice (Fig 5B). In this approach, we cannot track Mtb-specific CD4 T cells with tetramers due to the extremely low frequency of donor cells, so we assume that the transferred CD4 T cells that have upregulated CD44 are responding to the infection. The benefit of this approach, however, is that we can follow a very rare population of polyclonal PD-1 KO CD4 T cells in a WT recipient host. The Thy1.1^+^ PD-1 KO donor CD4 T cells contained a higher frequency of CD44^hi^ effectors compared to the CD45.1^+^ WT donor CD4 T cells (Fig 5C and 5D), consistent with the expected role of PD-1 in inhibiting T cell expansion. Similar to what we observed in intact PD-1 KO mice, a greater proportion of the PD-1 KO CD44^high^ donor CD4 T cells were localized in the parenchyma (Fig 5C and 5E), and there were increased frequencies of KLRG1^-^ CD4 T cells among the KO compared to WT donor effector cells in the same lungs (Fig 5C and 5F). As a control, we examined the naïve donor CD4 T cells derived from either WT or PD-1 KO mice and found them similarly localized to the lung parenchyma (Fig 5C and 5G). Importantly, the parenchymal donor effector CD4 T cells derived from PD-1 KO mice actively produced more IFN-γ than WT effectors as detected by DrxICS (Fig 5H). Together, these data indicate that the increased parenchymal CD4 T cell responses in PD-1 KO mice are not solely due to the elevated bacterial loads, and argue that PD-1 plays a cell-intrinsic role in inhibiting the generation of lung-homing CD4 T cells and their IFN-γ production in Mtb infection.

### PD-1-mediated inhibition of protective parenchymal CD4 T cells prevents IFN-γ driven mortality after Mtb infection

**Fig 6:**
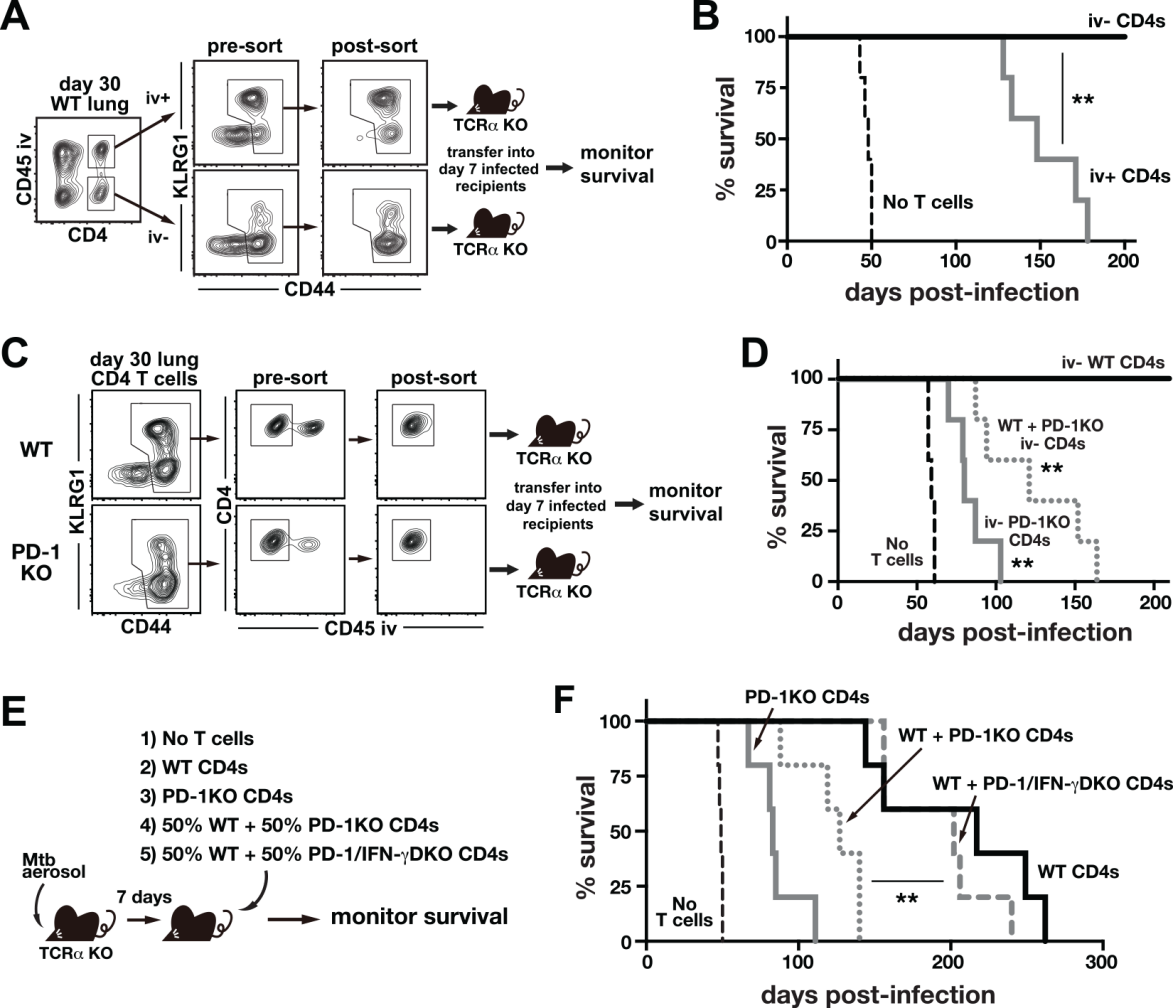
PD-1 KO CD4 T cells require IFN-γ production to drive early mortality after Mtb infection. (**A and B**) Parenchymal iv^-^ and intravascular iv^+^ effector CD44^hi^ CD4 T cells were FACS purified from the lungs of day-30 infected WT mice and adoptively transferred into TCRα KO mice that had been infected with Mtb 7 days previously (**A**) and mouse survival was monitored (**B**). **, *P*<0.001. (**C and D**) Parenchymal iv^-^ effector CD44^hi^ CD4 T cells were FACS purified from the lungs of WT and PD-1 KO mice on day 30 p.i., and adoptively transferred into day-7 infected TCRα KO mice (**C**) and mouse survival was monitored (**D**). Data are representative of two independent experiments (n = 5/group/experiment). **, *P*<0.002; compared to mice received iv^-^ WT CD4 T cells alone. (**E and F**) TCRα KO mice infected with Mtb 7 days earlier were reconstituted with WT, PD-1 KO or a mixture of WT and PD-1 KO or PD-1/IFN-γ double KO CD4 T cells (**E**) and survival was monitored (**F**). **, *P*<0.002 for WT + PD-1 KO (dotted red line) versus WT + PD-1/IFN-γ double KO (blue line). Data are representative of three independent experiments (n = 4-5/group/experiment).

We previously have shown that adoptive transfer of purified lung parenchymal CD4 T cells into T cell–deficient hosts resulted in greater reduction in bacterial loads at 4 weeks p.i. compared to transfer of intravascular cells [24]. To further examine the role of these CD4 T cell subsets in host resistance to Mtb infection, parenchymal iv-stain negative (iv^-^) or intravascular iv-stain positive (iv^+^) CD44^hi^ effector CD4 T cells were FACS purified from the lungs of WT mice on day 30 p.i. and adoptively transferred into TCRα KO mice that had been infected with Mtb 7 days prior to transfer (Fig 6A). Although both groups of recipient mice survived much longer than mice not receiving cells (Fig 6B), the recipients reconstituted with iv^+^ effector CD4 T cells all succumbed by ~day 180 p.i., whereas those reconstituted with iv^-^ effectors all survived for >210 days (Fig 6B). These data confirm that lung parenchymal Mtb-specific CD4 T cells are more protective against Mtb infection compared to intravascular CD4 T cells despite relatively low levels of IFN-γ production in vivo.

We next asked if the CD4 T cells located in the lung parenchyma of PD-1 KO mice were able to drive disease. To do so, iv^-^ CD44^high^ CD4 T cells were FACS sorted from the infected lungs of WT mice or PD-1 KO mice and adoptively transferred into TCRα KO mice that had been infected with Mtb seven days previously (Fig 6C). As shown in Fig 6B, iv^-^ parenchymal effector CD4 T cells derived from WT mice protected susceptible mice from the rapid mortality after Mtb infection (Fig 6D). By contrast, the parenchymal effector CD4 T cells derived from PD-1 KO mice accelerated the mortality of the reconstituted mice (mean survival time, 80 days). Moreover, mice reconstituted with a mixture of iv^-^ CD44^hi^ effector CD4 T cells from WT mice and PD-1 KO mice also rapidly succumbed to infection (mean survival time, 121 days), indicating that in the absence of PD-1 the parenchymal CD4 T cells that are normally protective against Mtb infection drive rapid mortality.

To test if increased IFN-γ production by the parenchymal PD-1 KO CD4 T cells was required to promote disease during Mtb infection, infected TCRα KO mice were reconstituted with WT or PD-1 KO CD4 T cells, or a mixture of WT and PD-1 KO or PD-1/IFN-γ double KO CD4 T cells (Fig 6E). Consistent with the previous finding [31], adoptive transfer of PD-1 KO CD4 T cells led to early mortality of the reconstituted mice, while WT CD4 T cells protected susceptible mice for a long period (mean survival time, 83 versus 217 days, respectively; Fig 6F). Furthermore, mice reconstituted with WT + PD-1 KO CD4 T cells died by day ~130 p.i., indicating that the presence of PD-1 KO CD4 T cells accelerated the mortality of reconstituted mice. Strikingly, mice reconstituted with WT + PD-1/IFN-γ double KO CD4 T cells survived as long as mice that received WT cells alone (mean survival time, 202 days; Fig 6F). Therefore, IFN-γ production by the co-transferred population of PD-1 KO CD4 T cells was required to drive the lethality. Taken together, these data demonstrate that the subset of lung-homing CD4 T cells that mediates host protection requires co-inhibition though PD-1 to inhibit IFN-γ–driven disease, highlighting how the same cells that are most protective against Mtb infection are potentially the most pathogenic if not properly regulated.

## Discussion

Here we used in vivo quantitative approaches to estimate the relative contribution of CD4 T cell-derived IFN-γ production to overall CD4 T cell-dependent control of Mtb infection. We compared the protective capacity of WT versus IFN-γ–overproducing CD4 T cells in different tissues. Our results show that IFN-γ from CD4 T cells accounts for ~30% of the total anti-bacterial effects of CD4 T cells in the lungs early in infection, and this relatively low contribution of IFN-γ is not explained by insufficient production of the cytokine by CD4 T cells. In fact, CD4 T cells that over-produce IFN-γ are less protective in the lungs and eventually drive lethal disease. In contrast, >80% of the protective effect of CD4 T cell responses in the spleen is due to their IFN-γ production, and the more IFN-γ is produced by CD4 T cells the more Mtb growth is suppressed in the spleen. We also found that the lung-homing, CXCR3^+^KLRG1^-^CX3CR1^-^ subset of CD4 T cells, that normally mediates the best control of pulmonary Mtb infection, requires inhibition through PD-1 to prevent the detrimental over-production of IFN-γ that leads to the death of the host. Clearly some IFN-γ production by CD4 T cells is essential for host survival of Mtb infection. However, the results presented here collectively show that CD4 T cell-derived IFN-γ has a predominant role in control of bacterial dissemination and/or suppression of Mtb growth at extra-pulmonary sites, but currently we cannot discriminate between these two possibilities. At the same time, production of this cytokine by CD4 T cells also has significant potential for inducing pulmonary pathology.

We have only sampled the spleen as a representative extra-pulmonary site of infection, and do not know the role of CD4 T cell-derived IFN-γ in other tissues. However, the hypotheses that CD4 T cell-derived IFN-γ is essential for control of either dissemination or replication in extra-pulmonary tissues, and that it may also be able to drive disease is consistent with observations on the role of IFN-γ in mycobacterial infection in humans and non-human primates. Individuals with CD4 T cell or IFN-γ deficiencies are well known to be highly susceptible to extra-pulmonary mycobacterial infections [40, 41], and Mtb infected cynomologous macaques depleted of CD4 T cells develop rapidly disseminating disease [42, 43]. Moreover, IFN-γ concentrations in BAL fluid have been found to positively correlate with severity of disease [44]. A few trials testing administration of recombinant IFN-γ to individuals with active TB have been completed. Several found no effects of recombinant IFN-γ treatment on the outcome of Mtb chemotherapy in individuals with drug-resistant infections [45–47], but one study was halted early due to increased mortality of TB patients receiving recombinant IFN-γ [48].

It is important to point out that the lethality we observed when CD4 T cells over-produced IFN-γ was likely due to an increase in the amount secreted by CD4 T cells on a per cell basis and not due to the total amount of IFN-γ found in the tissue nor the total number of cytokine producing CD4 T cells. Indeed, it is well known that increasing the numbers of Mtb-specific CD4 T cells through vaccination or other means results in better control of Mtb infection. However, our results suggest that the generation of highly-polarized T cells that secrete large amounts of IFN-γ on a per cell basis may be counter-productive for control of pulmonary disease in Mtb infection. In contrast, it has been shown that Yeti mice, which similarly to the ARE-Del mice over-produce IFN-γ due to alterations in their mRNA regulation, are highly resistant to *Listeria monocytogenes* and *Leishmania major* infection [49]. Therefore, enhanced disease is not a common outcome of increasing the per capita IFN-γ production by CD4 T cells responding to intracellular infections. Andersen and Urdahl have suggested that induction of highly polarized Th1 responses are not ideal for TB vaccines in part due to their poor longevity and inability to home into the lung [50, 51]. Our results support and extend this view. We should, however, emphasize that our estimation of the role for IFN-γ in CD4 T cell-mediated control of Mtb infection was restricted to the first ~6 weeks post-exposure, and we do not know the importance of CD4 T cell-derived IFN-γ for the durability of bacterial control.

The mechanisms underlying the tissue-specific effects of CD4 T cell-derived IFN-γ in control of Mtb infection are not clear. Our data are reminiscent of previous results showing that Mtb infection is exacerbated when TNF is either absent or over-produced [52, 53]. The same paradigm is also true for the IL-1 pathway, as mice deficient in IL-1 or mice that lack iNOS dependent inhibition of IL-1 production are both highly susceptible to Mtb infection [54–56]. The need to optimally balance inflammation to achieve the best bacterial control while not inducing pathology seems to be a major refrain in TB immunology. Indeed, our data are consistent with the damage-response framework Casadevall and colleagues have elegantly put forward, arguing that optimal host benefit during a response to infection is achieved when damage caused by both the microbe as well as the immune response itself are simultaneously minimized [57].

Our results also provide an example of how the class of immune response against a pathogen must be tailored to the tissue in which it occurs, as described by Matzinger [58]. The spleen much more readily accommodates CD4 T cell-mediated IFN-γ production compared to the lung. Mechanistically, there are several testable hypotheses to explain the tissue-specific effects of CD4 T cell-derived IFN-γ in Mtb infection. It has been shown that when macrophages and DCs harbor relatively high numbers of bacilli, high concentrations of IFN-γ induce the necrotic death of the infected macrophages rather than the containment of the bacilli [59, 60]. Although not addressed here, death of the most heavily infected myeloid cells in the lung due to the over-production of IFN-γ might contribute to the impaired control of the infection. The differential IFN-γ sensitivity of the lung and spleen may also represent the cell types present in those tissues. For example, lung epithelial cells have been shown to respond to IFN-γ and be important regulators of inflammation in Mtb infection [61], so high levels of IFN-γ production may induce qualitatively different responses by cell types unique to the tissue. Identification of the specific mechanisms downstream of the IFN-γ receptor that exacerbate Mtb infection in the lung might provide novel targets for host-directed therapy that aim to limit pathology-associated IFN-γ responses during TB.

There is great interest in the role that exhaustion of Mtb-specific CD4 T cells plays in the inability of the host to control Mtb infection. Given its major role in virus and tumor-specific CD8 T cell exhaustion, it was initially hoped that PD-1 dependent inhibition could likewise be targeted in chronic Mtb infection to boost T cell function and enhance bacterial control. However, PD-1 KO mice succumb to CD4 T cell-mediated pathology in Mtb infection [31], and here we identify the specific CD4 T cell subset controlled by PD-1, and implicate IFN-γ as an effector molecule responsible for the immunopathology in the absence of PD-1. These data are cause for concern about therapeutic strategies that boost CD4 T cell function in Mtb infection by targeting inhibitory receptors, now referred to as checkpoint blockade, and at least one report has noted TB reactivation associated with PD-1 blockade treatment of Hodgkin’s lymphoma [62]. However, it should be noted that PD-1 pathway deficient mice also succumb to LCMV infection [63]. Both Mtb and LCMV infected mice succumb near the peak of T cell clonal expansion, the first week in the case of LCMV and approximately the 5^th^ week for Mtb infection. In LCMV infection, the pathology is caused by CD8 T cell-mediated lysis of blood vascular endothelial cells [64], and in Mtb infection by IFN-γ–producing CD4 T cells. Although PD-1 blockade can be detrimental when peak numbers of activated effector T cells are present, blockade later in LCMV infection during the chronic phase results in enhanced viral control [63, 65, 66]. In the setting of Mtb infection, it has been found that PD-1 blockade at late stages of infection has no effect on bacterial loads and does not result in lethal pathology [23, 67]. It is not clear why PD-1 blockade in Mtb infection does not enhance immune responses, but it may be due to technical issues such as poor penetration of blocking antibodies into granuloma or compensation by additional inhibitory pathways that are upregulated as the infection progresses. Nonetheless, in both Mtb and LCMV infection the therapeutic window of checkpoint blockade opens after the peak of the T cell response when the risk of immunopathology decreases. It is possible that under antibiotic therapy PD-1 blockade during active TB may be safe. Therefore, further work is needed to determine if targeting checkpoint pathways could be developed as a safe and effective treatment strategy in Mtb infection. Perhaps other co-inhibitory receptors besides PD-1 that play less of a role in the control of IFN-γ production would be better targets. At the least, our data indicate that impaired IFN-γ production by exhausted Mtb-specific CD4 T cells is unlikely to explain the inability of the host to control Mtb infection. If CD4 T cell exhaustion does play a role in the poor control of Mtb infection, it may be due to the loss of other effector pathways besides IFN-γ.

While our data indicate that PD-1 inhibits the effector functions of Mtb-specific CD4 T cells, we also observed that PD-1 regulates the differentiation of CD4 T cells during Mtb infection. CD4 T cells in Mtb infected PD-1 KO mice displayed a dramatic decrease in KLRG1 expression and expressed lower levels of T-bet and CX3CR1 compared to WT CD4 T cells. Reduced KLRG1 expression and increased parenchymal localization of PD-1 KO CD4 T cells were also observed in our co-transfer experiments in WT recipient mice, indicating that the effect of PD-1 on CD4 T cell differentiation was intrinsic to the CD4 T cells and not secondary to elevated bacterial loads. We previously have shown that KLRG1^+^CX3CR1^+^T-bet^bright^ CD4 T cells are unable to migrate into Mtb infected lungs, and instead accumulate in the lung-associated blood vasculature. Collectively, our data suggest that in the absence of PD-1 dependent inhibitory signals, highly migratory CD4 T cells with a less differentiated phenotype are preferentially generated leading to an increase in lung parenchymal effector CD4 T cells, and once these cells get into the lung tissue, they over-produce IFN-γ. The role for PD-1 in promoting the generation of more differentiated Mtb-specific CD4 T cells was surprising, as it was expected that the increased signals into CD4 T cells in the absence of PD-1 dependent inhibition would enhance terminal differentiation. In fact, a recent report found that virus-specific PD-1 KO CD8 T cells in mice chronically infected with LCMV are driven into an even deeper state of terminal exhaustion [68]. Therefore, PD-1 plays a major role in regulating both the differentiation and effector functions of Mtb-specific CD4 T cells, but the precise role for PD-1 in the control of Mtb-specific effector CD4 T cell generation remains unclear.

Although several reports have shown that CD4 T cells can mediate IFN-γ–independent control of Mtb infection [32, 37, 69, 70], the relative contribution of these pathways in the overall control of Mtb infection was not quantified. Given the severity of infection in the absence of IFN-γ in animal models and the extreme susceptibility of humans with inborn errors of immunity in the IFN-γ axis to even environmental NTM infections, it is often stated that IFN-γ is the principal protective effector molecule produced by mycobacteria-specific CD4 T cells. Indeed, the host is extremely susceptible to early mortality during Mtb infection if CD4 T cells produce no IFN-γ. However, our data show that the mediators of the majority of bacterial control in the lung remain unknown, and indicate that identification of the pathways utilized by CD4 T cells to control pulmonary Mtb infection should be a top priority. The list of T cell effector molecules that have been shown to contribute to control of Mtb infection is not long. CD4 T cell-derived TNF has been shown to be important in host survival after intravenous [35] and aerosol [36] Mtb infection, but here we found that CD4 T cell-derived TNF plays a minor role in the lungs and spleens even compared to IFN-γ. It is possible that TNF in combination with IFN-γ mediates substantial CD4 T cell-dependent control, but future experiments such as we have described here are needed to directly quantify any potential synergy between these CD4 T cell-derived cytokines in control of Mtb infection. GM-CSF can also suppress the growth of Mtb in macrophages, and is another possible candidate, but GM-CSF derived from CD4 T cells has not been shown to contribute to protection to Mtb infection [71]. It seems likely that as yet unidentified effector pathways account for the largest part of T cell-dependent resistance to Mtb infection. Identification of novel anti-mycobacterial T cell effector molecules would provide mechanism based-correlates of protection to guide vaccine studies, and highlight potential targets for immunological interventions in TB that aim to selectively boost novel host protective pathways.

## Materials and Methods

### Ethics statement

Studies were performed in accordance with recommendation of the Guide for the Care and Use of Laboratory Animals of the National Institutes of Health (NIH). All experiments involving the use of animals were approved by the National Institute of Allergy and Infectious Diseases (NIAID) Animal Care and Use Committee.

### Mice and aerosol Mtb infection

C57BL/6 mice were purchased from Taconic Farms (Germantown, NY). B6.SJL (CD45.1) congenic, IFN-γ KO, TNF KO, TCRα KO and CD45.1 RAG1 KO mice were obtained through a supply contract between the NIAID/NIH and Taconic Farms. ARE-Del mice and have been described previously [33]. PD-1 KO mice [72] were crossed to IFN-γ KO mice in the NIAID animal facility. All animals (8–12 weeks old of sex matched) were housed at the Association for the Assessment and Accreditation of Laboratory Animal Care-approved BSL3 facility at the NIAID according to the National Research Council Guide for the Care and Use of Laboratory Animals. For Mtb infections, mice were exposed to ∼100 CFU of the H37*Rv* strain of Mtb with an inhalation exposure system (Glas-Col, LLC., Terre Haute, IN). Bacterial loads were measured in tissue homogenates by serial dilution on 7H11 agar plates supplemented with oleic acid-albumin-dextrose-catalase (Difco, Detroit, MI).

### Iv-staining and lung lymphocyte isolation

Mice were injected intravenously with 2.5 μg of fluorochrome-labeled anti-CD45.2 or anti-CD45 antibody, and after 3 min lungs were harvested [39]. Lungs were minced with scissors and then enzymatically digested for 45 min at 37°C in RPMI-1640 medium supplemented with 1 mg/ml Collagenase D (Roche-Diagnostics, Indianapolis, IN), 1 mg/ml hyaluronidase, 50 U/ml DNase I and 1 mM aminoguanidine (all from Sigma-Aldrich, St. Louis, MO). Digested lung was dispersed by passage through a 100 μm pore size cell-strainer and lung lymphocytes were enriched by 37% Percoll density centrifugation. For direct ex vivo IFN-γ staining, lungs were processed entirely in the presence of brefeldin A at a 1:1000 dilution (eBioscience, San Diego, CA).

### *In vitro* stimulation and flow cytometry

For T cell stimulations, cells were incubated with 5 μg/ml ESAT-6_1–20_ peptide for 5 h at 37°C in the presence of brefeldin A and 1 mM aminoguanidine. Cells were stained with various combinations of the following fluorochrome-labeled antibodies: anti-CD4 (RM4-4), CD44 (IM7), CD45 (IM7), CD45.1 (A20), CD45.2 (104), CD69 (H1.2F3), CXCR3 (CXCR3-173), CX3CR1 (polyclonal), Foxp3 (FJK-16s), ICOS (15F9), IFN-γ (XMG1.2), KLRG1 (2F1/KLRG1), PD-1 (29F.1A12), T-bet (eBio4B10), Thy1.1 (OX-7), and Fixable Viability Dye eFluor 780 purchased from BioLegend, eBioscience (San Diego, CA), BD Biosciences (San Jose, CA), and R&D Systems (Minneapolis, MN). I-A^b^ ESAT-6_4–17_ and I-A^b^ EsxG_46-61_ MHC tetramers were produced by the NIAID Tetramer Core Facility (Emory University, Atlanta, GA). For staining with MHC class II tetramers, cells were incubated with tetramer at 1:50 dilution in complete medium containing 10% FCS, 1 mM aminoguanidine and monensin (eBioscience) at a 1:1000 dilution for 1 h at 37°C prior to staining with surface antibodies. All samples were acquired on an LSRFortessa flow cytometer (BD Biosciences) and analyzed with FlowJo software (Tree Star, Ashland, OR).

### CD4 T cell isolation and adoptive transfer

Mtb-infected WT or PD-1 KO mice were intravenously injected with PE-labeled anti-CD45 antibody on day 30 p.i. Single cell suspensions were prepared from lungs of ~15 mice and pooled. Cells were stained with antibodies specific to CD4 (YTS177.9, Novus Biologicals, Littleton, CO), CD44, KLRG1 and Fixable Viability Dye, and then live intravascular or parenchymal CD44^hi^ CD4 T cells were sorted with FACSAria II (BD Biosciences) under the BSL3 condition. Greater than 97% purity of the sorted populations was achieved in all experiments. For survival experiments, ~4 × 10^4^ cells of each population were adoptively transferred intravenously into TCRα KO mice that had been infected with Mtb 7 days prior to transfer. For adoptive transfer of naïve CD4 T cells, cells were isolated from lymph nodes and spleens of WT, IFN-γ KO, ARE-Del, TNF KO, PD-1 KO or PD-1/ IFN-γ double KO mice using MACS magnetic beads and columns (Miltenyi Biotec. Auburn, CA). Naïve CD4 T cell purity was consistently >90% as determined by flow cytometry. RAG1 KO or TCRα KO mice infected 7 days previously were reconstituted with total 3 × 10^6^ cells of each indicated population. In additional experiments, MACS-isolated WT (CD45.1) or PD-1 KO (Thy1.1) naïve CD4 T cells were further purified to >97% purity by sorting, mixed at a 1:1 ratio and adoptively transferred (2 × 10^6^ total CD4 T cells) into Mtb-infected CD45.2 WT mice.

### Cytokine measurement in lung tissues

Levels of cytokines in sterile filtered lung homogenates were determined by Mouse IFN-γ and TNF ELISA kit (eBioscience) according to the manufacturer’s instructions.

### Statistical analysis

The statistical significance of difference between experimental groups was determined by Log-rank (Mantel-Cox) test, paired or unpaired Student’s *t* test using GraphPad Prism software (version 6). A *P*-value of <0.05 was considered significant.

For the determination of IFN-γ–dependent CD4 T cell-mediated control of Mtb growth in the tissues, CFU data were log_10_ transformed and area under the curve (AUC) for each CFU kinetic curve of unreconstituted mice, mice reconstituted with WT or IFN-γ KO CD4 T cells was calculated by the equations as shown in S1 Method. The following equation was used to calculate % CD4 T cell-mediated reduction in CFU that was IFN-γ–dependent:

$$\epsilon_{\gamma }=\frac{AUCunreconstituted–AUCreconstituted\;with\;IFN-\gamma KO\;CD4\;T\;cells}{AUCunreconstituted–AUCreconstituted\;with\;WT\;CD4\;T\;cells}\times 100\%$$ (1)

The calculations were performed for two independent experiments by averaging the log_10_ CFU per time point for each experiment. To calculate confidence intervals on the estimated efficacy we resampled CFU per time point for each experiment 10,000 times with replacement and re-calculated the efficacy using Eq (1). The statistical comparison between estimates of efficacy in the lung and spleen was done from 10,000 bootstrapped values [73].

## Supporting Information

S1 Method A mathematical analysis to estimate IFN-γ–dependent CD4 T cell-mediated control of Mtb growth in the tissues. (PDF)
