# Supplementary material for: CD4 T Cell-Derived IFN-γ Plays a Minimal Role in Control of Pulmonary Mycobacterium tuberculosis Infection and Must Be Actively Repressed by PD-1 to Prevent Lethal Disease
Source: PLoS Pathog. 2016 May 31;12(5):e1005667. doi: 10.1371/journal.ppat.1005667 (PMC4887085; doi:10.1371/journal.ppat.1005667)
Supplement: S1 Method — (PDF) [file ppat.1005667.s001.pdf]

Supplemental Information

Calculating IFN-γ-mediated suppressive efficacy of CD4 T cells using cumulative CFUs

In our experiments, Mtb cell numbers in the lung or spleen were continuously increasing over time. To estimate the relative efficacy at which IFN-γ-producing CD4 T cells suppress Mtb growth we used a simple mathematical model. In the model the bacterial population grows exponentially in the presence of immunity in accord with equation (see Figure S1):

$$\frac{\mathrm{d}B(t)}{\mathrm{d}t} = (g-k)B(t). \tag{S.1}$$

where g is the net accumulation rate of bacteria in the absence of immunity and k is the rate at which Mtb growth is suppressed by immunity. In general, both rates g and k could be (and are likely to be) time-dependent but our results were not strongly dependent on the assumption of the constant rates g and k (see below). Starting with B_0 bacteria, the number of bacteria at time t in the presence of immunity is

$$B(k,t) = B_0 \mathrm{e}^{(g-k)t}. \tag{S.2}$$

If rates g and k were time-dependent, then the difference $(g-k)$ should be treated as an average $\overline{g-k} = \overline{g} - \overline{k}$ over the time period $(0,t)$. The impact of the immunity on Mtb growth could be calculated in several different ways.

The most straighforward way was to estimate the rate of Mtb growth as the slope in the change in natural logarithm of Mtb counts over time. Then the efficacy of the immune response would be given by the difference in the slopes in the presence and absence of immunity, normalized by the Mtb growth rate in the absence of immunity. This would result in the estimate for immune response efficacy as k/g. However, because of noisy measurements of Mtb counts in lungs of individual mice this method led to relatively imprecise measurements of immune response-mediated suppression efficacy (results not shown). Another method was to calculate the total area under the curve describing bacterial counts over time. Area under the curve tended to be a more robust measure of growth because it was averaged over all the mice used in experiments. There are at least three different ways of how area under the Mtb counts curve could be calculated.

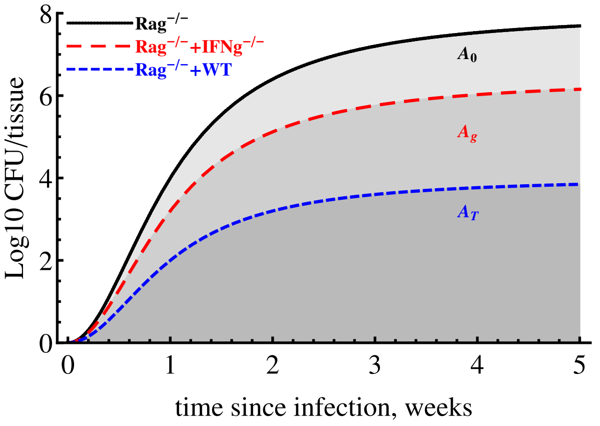

Figure S1: A cartoon representing Mtb growth in different conditions and how the relative efficacy of IFN-γ in suppressing Mtb growth can be calculated. Different lines denote Mtb growth in three different settings: in RAG1-deficient hosts (solid line), in RAG1-deficient hosts reconstituted with IFN-γ-deficient CD4 T cells (large dashing line), and in RAG1-deficient hosts reconstituted with WT CD4 T cells (small dashing line). Area under the curve for these three conditions can be calculated from experimental data and denoted as A_0, A_g, and A_T, respectively. The relative efficacy of IFN-γ in a tissue is given by the relative area under the log curve, $\epsilon_\gamma = \frac{A_g - A_T}{A_0 - A_T}$ (see eqns. (S.7)–(S.8) for derivation).

First was to calculate the average under the log-transformed Mtb counts. Using eqn. (S.2) this area-under-cuver (AUC) quantity is given by

$$A(k,t) = \int_0^t \log B(k,\tau)\mathrm{d}\tau = B_0 t + \frac{(g-k)t^2}{2}. \tag{S.3}$$

The relative efficacy of the immune response at reducing bacterial numbers is then calculated as the ratio of the difference in cumulative number of CFUs over cumulative CFU in the absence of immunity for the infection of duration T

$$\epsilon_A = \frac{A(0,T) - A(k,T)}{A(0,T)} = \frac{\frac{gT^2}{2} - \frac{(g-k)T^2}{2}}{B_0 T + \frac{gT^2}{2}} = \frac{k}{g + 2B_0/T}. \tag{S.4}$$

Efficacy $\epsilon_A \approx \frac{k}{g}$ if the ratio B_0/T is sufficiently small. Note that extending the initial model (eqn. (S.1)) with time-dependent rates g and k could still allow to calculate the average efficacy of the immune response, $\epsilon = \bar{k}/\bar{g}$ with $\bar{g}$ and $\bar{k}$ being the average growth and suppression rates over the infection.

Second was to calculate the total cumulative number of bacteria N and and then compare the logarithms of cumulative numer in the absence or presence of immunity. The cumulative number of bacteria by time t is given by

$$N(k,t) = \int_0^t B(k,\tau)\mathrm{d}\tau = \frac{B_0}{g-k}\left(\mathrm{e}^{(g-k)t} - 1\right). \tag{S.5}$$

and the relative efficacy of the immune response in suppressing bacterial accumulation is then

$$\epsilon_N = \frac{\log N(0,t) - \log N(k,t)}{\log N(0,t)} = \frac{kT + \log\left(\frac{g}{g-k}\right)}{gT + \log B_0 - \log g}. \tag{S.6}$$

Efficacy $\epsilon_N \approx \frac{k}{g}$ if T is sufficiently large and $g \gg k$. This method requires more assumptions and is not easily extentable to time-dependent rates g and k (results not shown).

Third and final method was to calculate the efficacy by using the cumulative number of bacteria over the course of infection, $\epsilon_{N_t} = \frac{N(0,t)-N(k,t)}{N(0,t)}$. This method did not lead to an accurate estimate of the relative efficacy of the immune response at suppressive bacterial growth (results not shown). Taken together, out of three methods involving analysis of the total number of bacteria over the course of infection the first method (eqn. (S.4)) was the most robust method to estimate the impact of immunity on bacterial growth.

To calculate the efficacy of CD4 T cell-derived IFN-γ in suppression of Mtb growth in the lung and spleen we estimated the total area under the log-transformed CFUs over the course of infection (eqn. (S.3)). As we performed three different types of experiments, we calculated three different AUCs (Figure S1): A_0 was the cumulative Mtb counts in the absence of T cells, A_g was the cumulative Mtb counts in RAG1-deficient hosts reconsitituted with IFN-γ-deficient CD4 T cells, and A_T was the cumulative Mtb counts in RAG1-deficient hosts reconsituted with wild-type CD4 T cells. By dividing the total suppression efficacy of CD4 T cells (k) into IFN-γ-dependent (k_γ) and IFN-γ-independent efficacy (k_i, so $k = k_\gamma + k_i$), a simple model (eqn. (S.1)) predicted the following expressions for cumulative Mtb counts

$$\begin{aligned} A_0 &= A(0,t) = B_0 t + \frac{gt^2}{2}, \\ A_g &= A(k_i,t) = B_0 t + \frac{(g-k_i)t^2}{2}, \\ A_T &= A(k,t) = B_0 t + \frac{(g-k)t^2}{2}, \end{aligned} \tag{S.7}$$

and the relative efficacy of IFN-γ in suppressing Mtb growth after a simple algebra is given by (see Figure S1)

$$\epsilon_\gamma = \frac{A_g - A_T}{A_0 - A_T} = \frac{k_\gamma}{k}. \tag{S.8}$$

Note that because the denominator in eqn. (S.8) involves the difference in two areas under the curve, the calculation of efficacy is independent of the assumption of a small product $2B_0/T$ as this term disappears in the difference. Thus, experimental ratio of differences in AUC given eqn. (S.8) provides a reasonable estimate of the relative efficacy of CD4 T cell-derived IFN-γ in suppressing Mtb growth. Therefore, it was used in the main text.

List of Figures

S1 A cartoon representing Mtb growth in different conditions and how the relative efficacy of IFN-γ in suppressing Mtb growth can be calculated. Different lines denote Mtb growth in three different settings: in RAG1-deficient hosts (solid line), in RAG1-deficient hosts reconstituted with IFN-γ-deficient CD4 T cells (large dashing line), and in RAG1-deficient hosts reconstituted with WT CD4 T cells (small dashing line). Area under the curve for these three conditions can be calculated from experimental data and denoted as A_0, A_g, and A_T, respectively. The relative efficacy of IFN-γ in a tissue is given by the relative area under the log curve, $\epsilon_\gamma = \frac{A_g - A_T}{A_0 - A_T}$ (see eqns. (S.7)–(S.8) for derivation). 2
